# Modern Sociology

**Published:** 1896-11-28

**Authors:** 


### Modern Sociology.

### MISTAKEN IMPRESSIONS OF PRISON LIFE.—IV.

The sentence of hard labour during imprisonment, which is almost always awarded to ordinary criminals, is one of the points connected with the administration of justice, on which popular feeling is often somewhat strongly aroused. Hard labour has an unpleasant sound, and undoubtedly it is in a great measure an unpleasant experience, and yet it is a fact that many prisoners feel themselves decidedly aggrieved, when, for reasons carefully weighed in the regular course of prison routine, they are sometimes deprived of the labour assigned for the occupation of most of their daylight hours. This infringement of the regular rule may take place from a variety of different causes. Solitary detention in the cell for longer or shorter periods is one of the punishments dealt out to those who have been guilty of misconduct; or, again, if a prisoner is suffering from some indisposition not sufficiently serious to necessitate his removal to the infirmary the doctor often orders him to be kept in bed, or, perhaps, only to remain quietly in his cell for two or three days, where he is supplied with any remedies that may be required, and also with such an improved scale of diet as the medical officer considers suitable for his case. Yet even when inaction and silence take this beneficent form of repose for the good of his health, the prisoner is apt to resent the loss of his hard labour, as if some luxury had been taken from him. We make this statement on the testimony of numberless prisoners who have discussed the subject with us, and explained their own views respecting it in the clearest manner. They do not patronise hard labour on its own merits. Viewed apart from its alternative it is a detestable infliction, from which they would willingly shake themselves free; but it is incomparably preferable in their minds to the monotonous idleness of their own cells, where a Bible and Prayer-book are their sole means of occupation—not, it is to be feared in many instances, a species of literature which commends itself to their favour, even when they are able to read, which many are not, in spite of the School Board. Thus the slow hours have to be dragged through wearily in complete inaction. Very few of them are of the type who could say "My mind to me a kingdom is"; they are not accustomed to exercise the power of thought on anything but their immediate surroundings, and there is not much to think about in their four stone walls and narrow slit of a window high up near the ceiling, Many of those who, lying on their small beds, think with longing of their ordinary hard labour, have been, when masters of themselves outside, quite incorrigibly idle—lazy rogues who would rather starve than do a stroke of work, or, at least, infinitely prefer to get a scanty nourishment by begging, rather than obtain it by honest toil in the fields or elsewhere. The anomaly of their desire for the hard labour of the gaol is simply due to the very great difference which exists, between idleness as enjoyed in freedom and that ordered for them by prison regulations. To lie on his back on the grass in fine summer weather, or to loll on a bench outside a public-house and converse with the passers-by, is as pleasant an experience for the lazy vagabond as rigid confinement to a prison cell is distinctly the reverse, so that we need not wonder he considers hard labour, probably in the open air, preferable to that peculiar form of idleness. The conditions of labour appointed to criminals are not the same in all our judicial establishments, and this fact has probably given rise to some misconceptions on the subject. In the convict prisons to which those sentenced to penal servitude are consigned, there is no crank or treadwheel or oakum picking allowed, but these are still employed in the local prisons where all convicts pass their preliminary time of detention. Every criminal condemned to penal servitude, for whatever period, has to undergo first nine months' solitary confinement in the prison belonging to the town where his trial has taken place, and, therefore, during that time he must share the species of labour awarded to those who will complete their term of imprisonment within the local gaol. A considerable portion of that work is quite unexceptional, such as making mail-bags for the Post Office, and supplying what is necessary for the maintenance or repair of all that is required in every way within the prison itself. But it is much to be hoped that the absolutely useless and aimless labour of the tread-wheel and crank may soon be altogether abolished, or at least made by machinery to accomplish some definite purpose, such as grinding corn or pumping water. At present the treadwheel produces no other result than the fatigue of the prisoner. For six hours each day except Sunday he has to mount these revolving steps which lead nowhere and accomplish nothing; he has to go on unremittingly for a quarter of an hour, then is allowed five minutes' rest on a bench placed below. That short period of breathing time past, he mounts again for another quarter of an hour, when a second five minutes' respite follows, and so on through the whole six hours; and he knows all the time that the vigour and energy he is compelled to expend on this hopelessly aimless proceeding is finally and totally wasted. It certainly seems an outrage on the beautiful mechanism of the human body that all its wonderful powers of muscular strength and complex action should be bestowed on what has emphatically been termed grinding the wind. There is, however, we trust, good hope that this senseless infliction, an utter waste of time and strength, will not long remain a part of the prison code. We believe it is even now under consideration that the crank and treadwheel should be made to accomplish very excellent work by means of machinery in various ways, and there is no doubt that if the prisoners engaged on it could know that they were usefully employed for the ultimate benefit of their fellow creatures, it would remove the galling sense of utterly wasted labour which weighs on them now, as they go through their weary round of useless drudgery. We have good reason to know that the details of criminal labour, as well as all other items of prison rule, are now most carefully and humanely considered by the Prison Commissioners, and measures for improvement in many ways are already being enacted. For instance, in the month of June in the present year the picking of oakum by the female prisoners was finally abolished; it is still retained in local prisons for the men, but it is employed as little as possible; they are taught various trades where it can be done, and useful work is found for them in the prison itself and in the grounds surrounding it.

